# X-ray near-field multi-slice ptychography for in-situ imaging

**DOI:** 10.1038/s41598-025-15610-8

**Published:** 2025-09-01

**Authors:** Sina Röper, Karolina Stachnik, Jonas Voss, Sarah-Alexandra Hussak, Mattias Åstrand, Lukas Grote, Felix Wittwer, Martin Seyrich, Sven Niese, Peter Gawlitza, Ulrich Vogt, Christian G. Schroer, Dorota Koziej, Andreas Schropp

**Affiliations:** 1 Center for Hybrid Nanostructures, Institute for Nanostructure and Solid-State Physics, University of Hamburg, Luruper Chaussee 149, 22761 Hamburg, Germany; 2 Center for X-ray and Nano Science CXNS, Deutsches Elektronen-Synchrotron DESY, Notkestraße 85, 22607 Hamburg, Germany; 3 Department of Applied Physics, Bio-Opto-Nano Physics, KTH Royal Institute of Technology, Albanova University Center, 106 91 Stockholm, Sweden; 4 NERSC, Lawrence Berkeley National Laboratory, Berkeley, CA 94720 USA; 5 Deutsches Elektronen-Synchrotron DESY, Hamburg, Germany; 6 AXO DRESDEN GmbH, Gasanstaltstraße 8b, 01237 Dresden, Germany; 7 Fraunhofer Institute for Material and Beam Technology (IWS), Winterbergstrasse 28, 01277 Dresden, Germany; 8 Helmholtz Imaging, Deutsches Elektronen Synchrotron DESY, Notkestraße 85, 22607 Hamburg, Germany

**Keywords:** Imaging techniques, Scanning probe microscopy, Phase-contrast microscopy, Nanoparticles, Structural properties

## Abstract

In-situ imaging of chemical reactions can provide valuable insight into nanoparticle growth and structural evolution. Hard X-ray imaging is an excellent tool for this purpose, as it combines high spatial resolution with high penetration depth, allowing for realistic reaction environments. While far-field ptychography is a well-established method at synchrotron radiation sources, its near-field analog has received less attention. In this work we show that near-field multi-slice ptychography is a competitive method for high-resolution in-situ imaging by studying the formation of gold nanocages via galvanic replacement. Our work extends near-field X-ray ptychography to sub-50 nm spatial resolution by using multilayer Laue lenses. These high numerical aperture optics enable to distinguish sample layers that are separated by less than 100 µm by multi-slicing techniques.

## Introduction

Shape, size and anisotropy of nanoparticles play a significant role in determining their physical properties. They can influence optical, electronic, catalytic, and magnetic properties as well as biological interactions^1,2^. Insight into the morphological changes of particles during chemical reactions is invaluable to precisely tune the synthesis to achieve specific surfaces and shapes. The detailed characterization of such processes is challenging due to the small length scales in the nanometer range requiring high spatial resolution techniques. Here, characterization tools are mainly based on electron or X-ray microscopy that are able to image single nanoparticles due to their short wavelength.

Transmission electron microscopy (TEM) provides both high spatial and temporal resolution. It was applied to study the growth and shape evolution of nanoparticles in solution in situ for several materials^3–7^. TEM was for example used to observe the Kirkendall effect in the galvanic replacement reaction of silver nanocubes with gold^8^. However, TEM has severe limitations with respect to possible reaction environments due to the small penetration depth of electrons in matter. This limits significantly the reaction volume, the temperature or the pressure range that can be applied. Hard X-ray imaging, in contrast, is an excellent tool to probe a larger area of bulky samples and sample environments. Electron and X-ray microscopy are ideally used as complementary techniques. A good example is a study by Han et al. on the nucleation and growth of 3D transition bimetallic nanocrystals with in-situ TEM and spectroscopic ptychography^9^.

For high-resolution imaging, ptychography has become the workhorse at synchrotron radiation sources. However, most of the research has focused on far-field ptychography (FFP) so far. Nevertheless, near-field ptychography (NFP) has some key advantages as, e.g., it requires fewer scan points to image a large region with high spatial resolution^10^.

It has been shown that the resolution in coherent diffractive imaging is inherently limited by the applied dose^11,12^. In their comparative study, Du et al. found no significant difference between near-field and far-field techniques in this regard^13^. For both, the photon fluence on the sample sets the limits for spatial resolution. However, NFP is far less sensitive to partial coherence than FFP^14,15^. This allows NFP to use larger fractions of the X-ray beam, resulting in a greater number of photons delivered to the scanned area within a comparable time unit. Considering the relationship between dose and resolution, NFP has the potential to offer faster scans for a given resolution and field of view.

For in-situ imaging, however, the limiting factor is often not only the available photon flux, but the dose that a sample system can withstand before beam damage becomes apparent. Beam-damage effects can significantly disturb the study of chemical reactions under in-situ conditions. Several factors influence the severity of radiation damage to the sample, such as sample composition, solvents, temperature, pH, photon energy, beam size, dose or dose rate^16,17^. The sensitivity of a sample to radiation damage is very sample dependent and so are the measures that can be taken to reduce the degradation. The state of the art for X-ray studies of biological samples is cryocooling or the use of free-radical scavengers^18–20^. This is often not possible with in-situ or operando imaging, where specific temperatures and chemical environments are required to emulate realistic conditions.

NFP utilizes larger illumination sizes than FFP that significantly reduce the dose rate. The dose can also be further decreased by performing NFP imaging at higher X-ray energies^21^. The typical decrease of spatial coherence at higher photon energies at synchrotron radiation sources^14,22^ is then compensated by the weaker coherence requirement of NFP. This makes near-field ptychography a very compelling method for in-situ imaging of chemical reactions. While the advantages listed above also apply to in-line holography, NFP has less stringent illumination requirements. In fact, the use of a highly structured illumination improves the reconstruction in NFP^10^.

In our first demonstration of NFP for imaging nanoparticle growth, we use a reaction container with a size that exceeds the depth resolution of the microscope. We address this problem by exploiting near-field multi-slice ptychography, which enables imaging of optically thick objects by distinguishing between different sample layers. Combining multi-slicing and NFP pose the particular challenge that the distances between the slices and their number must be known precisely, which has been addressed by an iterative refinement and was demonstrated experimentally for optical wavelengths^23^. A first demonstration of near-field multi-slice ptychography with X-rays was reported^24^ and the method was refined for imaging in the range of optical wavelengths^25^. In order to obtain an optimal resolution in the layer of interest, a high depth and lateral resolution of the imaging system is required, which can be achieved with high numerical-aperture lenses. At the same time, thick samples require the use of hard X-rays, where absorption is significantly lower. For this energy range ($>$ 10 keV), multilayer Laue lenses (MLLs) provide the highest numerical aperture and the smallest focus size. In far-field multi-slice ptychography, MLLs enabled imaging with a slice thickness of 10 µm^26^.

In this work, we present in-situ near-field multi-slice ptychography with MLLs of the galvanic replacement reaction of cuprous oxide with gold. In a first ex-situ experiment, we perform multi-slice NFP on a two-layer nanoparticle sample. In two in-situ studies, we demonstrate the capabilities of NFP for low dose rate and high spatial resolution imaging.

## Near-field multi-slice ptychography

Near-field ptychography is a scanning phase-contrast imaging technique, which recovers the illumination and the sample transmission function from overlapping scan positions. Multi-slice reconstructions extend the capabilities of ptychography to the imaging of optically thick specimens. It was initially used in FFP^27^ and has recently been introduced for the near-field regime^24^. Multi-slice ptychography enables multiple layers of a 3D sample to be recovered.

The minimum thickness of layers distinguishable in multi-slice ptychography is described by the depth of field of the imaging system. In general, the depth of field (DOF) in ptychography is defined as:

1 $$\begin{aligned} {DOF} = c \cdot \frac{\delta _r^2}{\lambda } \end{aligned},$$

where $\delta _r$ is the lateral resolution, $\lambda$ the wavelength of the illumination, and *c* a constant reported to be between 1.0 and 5.2^28–30^. Stockmar et al. defined $c:=1.0$ for NFP^28^. Hu et al. define the depth resolution for near-field multi-slice ptychography based on raytracing as:

2 $$\begin{aligned} {DOF} = \frac{\delta _r \cdot (z_1+z_2)}{D} \end{aligned},$$

where $z_1+z_2$ is the propagation distance between the focus of the cone beam and the detector and *D* is the beam diameter on the detector^24^. For small angles, as generally valid for X-ray optics, one can rewrite Eq. (2) with the numerical aperture as $\textit{NA}\approx 0.5\cdot D/(z_1+z_2)$. Defining $NA$ in terms of the Abbe criterion is identical to Eq. (1) with $c=1.0$, so this again matches the definition by Stockmar et al.^28^. The achievable resolution in NFP is slice dependent since the pixel size of each slice depends on the individual focus-to-slice distance. Therefore, the DOF in NFP, as defined by Eqs. (1) and (2), is also slice dependent.

Although NFP is a lensless imaging technique that does not use an objective lens, the achievable depth resolution as described in Eq. (2) is directly related to the numerical aperture of the lens placed upstream of the sample. To achieve superior lateral and depth resolution, high-*NA* lenses should be used.

**Fig. 1:**
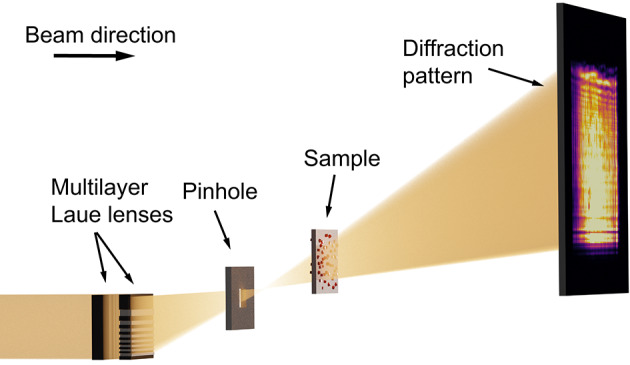
Schematic of the experimental setup. The X-ray beam is focused by a set of two MLLs to a focal spot of 30 nm (h) $\times$ 24 nm (v) (FWHM) in horizontal and vertical direction, respectively. A pinhole between the lenses and the focal spot acts as an order sorting aperture and cleans the beam. The sample is positioned at a distance between 0.63 mm and 3.00 mm downstream of the focus and scanned laterally across the beam. For each scan point, a diffraction pattern is recorded on a photon-counting detector at a distance of 3290 mm behind the sample. Image is not to scale.

Many in-situ studies require bulky reaction environments. For this reason, a high penetration depth of the illuminating beam is required, which can be achieved by using hard X-rays. MLLs are the optics type with the highest *NA* in the hard X-ray range and were therefore utilized for this setup. In our experiment, we set the photon energy to $E =$ 18 keV. A set of MLLs for horizontal and vertical focusing created a focal spot of 30 nm $\times$ 24 nm (see supplementary material Fig. S10). The MLLs were illuminated fully coherently for the in-situ series shown in Fig. 3. For the other measurements prefocusing compound reflective lenses located 54 m upstream of the MLLs were used. These reduced the spatial coherence length at the MLL position to 15 µm in the horizontal and 85 µm in the vertical direction and led to a partially coherent illumination. In near-field imaging, coherence over the first Fresnel zone width would be sufficient^31^ and the coherence length for all measurements is well above this limit. As shown schematically in Fig. 1, the focused beam was cleaned from other diffraction orders by a rectangular pinhole. The sample was positioned on a piezo scanner between 0.63 and 3.00 mm downstream of the focal plane and diffraction patterns were recorded at a distance of 3.29 m behind the sample by an Eiger X 4M in-vacuum detector. Further details of the setup can be found in the Methods.

The scans were reconstructed using the ePIE^32^ algorithm for the single-slice case and the 3PIE^27^ algorithm for the multi-slice case. In the reconstruction, the wavefield is propagated between the slices and between the sample and the detector using a Fresnel propagator. Details of the reconstruction parameters for each scan are tabulated in the supplementary material. In contrast to FFP, NFP is modeled with a cone beam geometry. For efficient image reconstruction, the cone beam must be converted to a parallel-beam geometry, which is done by applying the Fresnel scaling theorem^33^. The pixel size and the propagation distance of each slice are scaled accordingly. This step requires the knowledge of the distance between the focus and the sample, measured during the experiment. In this work, the focus-to-sample distance and the distances between slices were refined interactively by performing reconstructions with the aforementioned distances varied by $\pm 10\%$ around the expected value. The values that produced a flat phase profile for the illumination and visually good results for the slices were chosen for the final reconstruction. The adjustments in the 3PIE algorithm can be found in the supplementary material. In our experiment, the divergent MLL-beam had a size of 11.3 mm $\times$ 12.6 mm on the detector, with a propagation distance of 3.29 m between sample and detector. Using Eq. (2), it is found that the depth resolution is $DOF\approx 275 \cdot \delta _r$. At a source-size-limited lateral resolution of $\delta _r=$ 30 nm the achievable depth resolution with our setup is therefore $DOF=$ 8 µm.

### Capabilities for ex-situ imaging

**Fig. 2:**
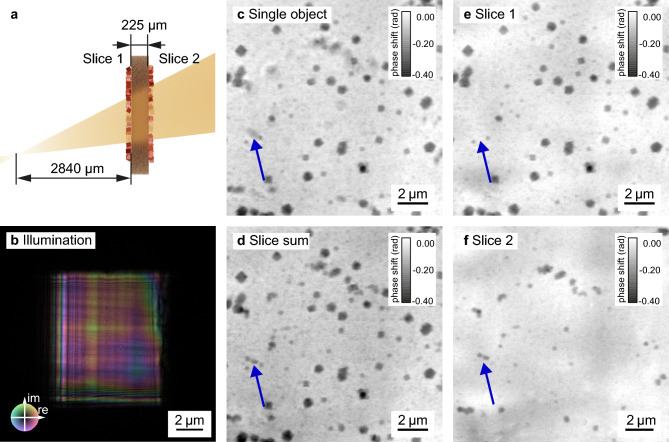
Near-field multi-slice ptychography of ex-situ cuprous oxide nanoparticles deposited on both sides of a polyimide foil. (**a**) schematic of the sample placed 2840 µm downstream of the focus of the X-ray beam with the two particle layers separated by a 225 µm-thick polyimide foil. (**b**) shows the corresponding reconstructed illumination with a size of 8.5 µm $\times$ 10 µm. (**c**) reconstruction of the scan assuming a single optically thin object. (**d**) summed phase of the multi-slice reconstruction shown in (**e**) first slice (upstream) and (**f**) second slice (downstream). The particles on the downstream side of the foil (slice 2) appear blurred in the single slice reconstruction (see e. g. the blue arrow), while they are well resolved in the reconstruction of the second slice and in the sum of the slices.

We first characterize our method by imaging cuprous oxide nanoparticles synthesized ex situ on both sides of a 225 µm-thick polyimide foil, as conceptually illustrated in Fig. 2a. Details of the image acquisition are described in the Methods—Scanning parameters section. The reconstructed illuminating beam (Fig. 2b) shows a relatively flat phase profile (indicated by the colors), as it is expected in near-field imaging without a diffuser^10^. The grid-like structure visible in the amplitude of the illumination (indicated by the brightness) is caused by the layered structure of the multilayer Laue lenses. Structure in the illumination is in general beneficial for a robust reconstruction in ptychography^34,35^ and even more important in NFP. For efficient phase retrieval, NFP relies on a structured illumination^10,15^. The structure is conventionally realized with a diffuser, such as sand paper, positioned in front of the lens. The use of MLLs, whose slight imperfections produce an inherently structured wavefield, made a diffuser unnecessary in our work.

For the multi-slice reconstruction, the polyimide foil is a model specimen with two distinct planes of cuprous oxide cubes on either side of the foil, slice 1 and slice 2, as shown in Fig. 2a. A conventional NFP reconstruction of the foil, treating the object as optically thin, is shown in Fig. 2c. The features on the upstream side of the foil are well resolved and sharp, while the cuprous oxide cubes on the back side appear blurred (see blue arrow in the figure). This indicates that the single-slice approximation for the object is not sufficient to model the thicker sample. To resolve both sides of the foil, beam propagation effects between the different layers must be taken into account. With multi-slice NFP, the individual slices can be sharply recovered. Slice 1 (Fig. 2e) shows the particles located on the upstream side of the foil and slice 2 (Fig. 2f) the nanocubes on the downstream side. The pixelwise sum of the two slices, shown in Fig. 2d, exhibits sharp edges for all features of both layers. Due to the cone-beam geometry, the effective pixel sizes are not the same for the two object slices. The pixel size for slice 1, which is closer to the focus, is 64.7nm and for slice 2 it is 69.8nm. To create the pixelwise slice sum in Fig. 2d as a parallel projection, we scaled the pixel size of slice 2 to match the pixel size of slice 1 using the scikit-image^36^ resize function.

Background fluctuations (brighter and darker areas without any sharp edges) are apparent in the reconstructions of the slices and cancel out in the sum of the two. These low-frequency image artifacts are often present in multi-slice ptychography^30,37^, typically caused by the short propagation distance between the slices. The maximum width between two points in one plane, for which interference effects can be observed after propagation over an effective distance $z_\textrm{eff}$, can be estimated by the first Fresnel zone radius $r_F$^38^:

3 $$\begin{aligned} r_F=\sqrt{\lambda \cdot z_\textrm{eff}} \end{aligned},$$

where $\lambda$ denotes the X-ray wavelength. This effectively limits the lowest spatial frequency that can be recovered for a given propagation distance.

In the multi-slice reconstructions, the scattering of the polyimide foil was neglected, and the wavefield propagated between the two slices. In reality, though, the polyimide foil introduces a phase shift. However, this effect is negligible at the used photon energy for a homogeneous foil of the given thickness. Applying Eq. (2) with a lateral resolution of $\delta _r=$ 102.6 nm, the DOF for this scan was equal to 27.5 µm while the reconstructed slices were separated by 225 µm, which is well above the DOF limit. Here, we demonstrated multi-slice NFP on an ex-situ model sample; in the next step we show its capabilities for in-situ imaging of chemical reactions.

### In-situ imaging of the galvanic replacement reaction of cuprous oxide nanocubes with gold

**Fig. 3:**
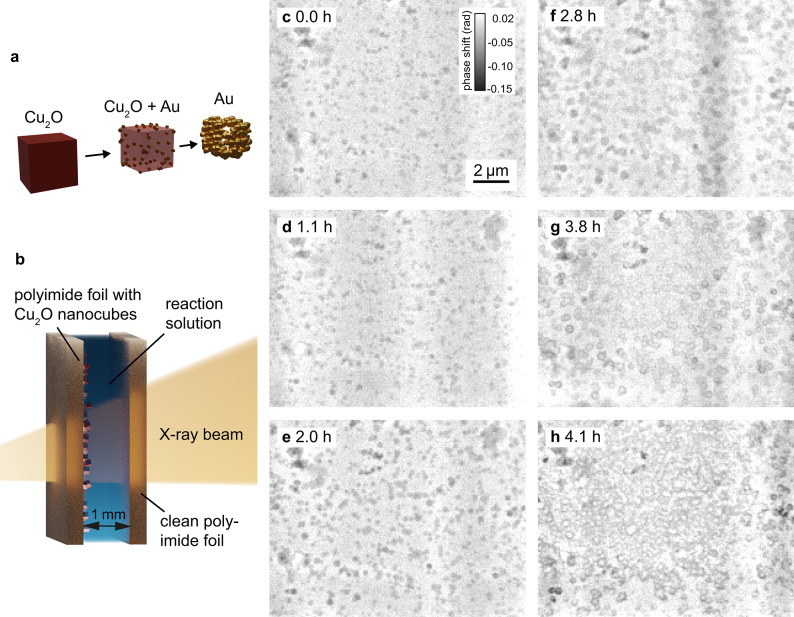
In-situ near-field multislicing ptychography of cuprous oxide nanocubes undergoing a galvanic replacement reaction. (**a**) Schematic of the galvanic replacement reaction. Gold particles form on the surface of the cuprous oxide cubes, $\hbox {Cu}^{2+}$ dissolves over the course of the reaction and hollow gold nanocages form (adapted from^39^). (**b**) Schematic of the reaction container. A polyimide foil with pre-synthesized cuprous oxide and a clean foil encapsulate the reaction solution with the gold precursor. (**c**)–(**h**) In-situ galvanic replacement reaction over 4.2 h. Multi-slice near-field reconstructions of the upstream window (phase shift).

The galvanic replacement reaction of cuprous oxide nanocubes with gold acts as a model reaction for in-situ multi-slice NFP. We mounted our reaction cell for in-situ imaging of nanoparticle growth^39^ at a distance of 3.0 mm behind the focal plane. The polyimide foil with the cuprous oxide cubes is placed on the upstream side of the reaction container. The downstream window is an empty polyimide foil, which is separated by 1 mm from the upstream window using a PTFE-frame between them. The resulting reaction container is enclosed in a metal casing (for photographs of the cell see supplementary material Fig. S5). The reaction solution containing the gold precursor is injected between the two polyimide windows. The pre-synthesized cuprous oxide nanocubes are attached to a polyimide foil and, over the course of the reaction, the cuprous oxide is replaced by gold, as schematically shown in Fig. 3a,b. The reaction conditions are described in the Methods—Synthesis procedure and details can be found in Grote et al.^39^.

The in-situ series of the galvanic replacement reaction of $\hbox {Cu}_2 \hbox {O}$ with Au was measured over 4.2h. A single ptychographic scan spanned a field of view of 10 µm $\times$ 10 µm and required a total scan time of 9.6 min. The reconstruction parameters can be found in the supplementary material Table S2.

For this in-situ series, the *DOF* was 21 µm and the two reconstructed slices were separated by 1 mm. Scattering by the reaction solution was neglected and the distance between the two windows was modeled with a free-space propagator. In the post-processing step, a high-pass filter was applied to the reconstructed phase images of the upstream window to reduce low-frequency artifacts. We used the scikit-image^36^ Butterworth filter with a cutoff frequency ratio of 0.018.

At the beginning of the reaction at 0.0 h (Fig. 3c), the upstream polyimide foil was covered with $\hbox {Cu}_2 \hbox {O}$ cubes with an edge length of 150 nm to 250 nm (see Supplementary Fig. S7). The reaction solution containing the gold precursor (20 mM $\hbox {HAuCl}_4$) was slowly injected into the cell with a syringe pump. In the first phase of the reaction, Au particles formed on the surface of the nanocubes. In the second phase, the Au particles grew larger and fused together, $\hbox {Cu}_2 \hbox{O}$ was further oxidized and dissolved, and the cubes became less dense in their centers. The final stage of the reaction was the formation of hollow Au nanocages, similar like observed in our FFP experiments reported previously.^39^

Between 0.0 h and 2.8 h (Fig. 3c–f), a growth of the particles could be observed. A beam dump between 3.0 h and 3.8 h after the start of the reaction did not allow data acquisition during this period. In the first scan after the beam dump (at 3.8 h, Fig. 3g), the particles appeared hollow and in the subsequent scans the hollow structures seemed to connect. The growth in the first phase can be attributed to Au particles forming on the $\hbox {Cu}_2 \hbox {O}$ surface. The galvanic replacement reaction progressed during the beam dump and at 3.8 h after the start of the reaction the hollow cages resembled the expected final result. The later Au deposition (Fig. 3h) can be attributed to beam damage. This is also evidenced by Au deposition on the downstream window of the reaction container (see Fig. S8 in the supplementary material), which is not the case in comparable ex-situ laboratory experiments.

For this in-situ series, the spatial resolution determined by Fourier-ring correlation was between 88 nm and 129 nm (see supplementary material Fig. S2), which is comparable to the spatial resolution achieved in a similar experiment with FFP^39^. The average dose per projection was 0.36 MGy with a dose rate of 1.28 kGy/s, which corresponds to a reduction in dose by a factor of 2.5 for a given field of view and a reduction of the dose rate by more than two orders of magnitude compared to the FFP results. The dose and dose rate were calculated according to the procedure reported by Grote et al.^39^. The significantly reduced dose rate is a result of the large illumination. However, the cumulative dose is comparable and, therefore, so is the spatial resolution.

The growth and formation of the hollow cages was clearly observed. This study demonstrates the competitiveness of NFP for in-situ imaging with reduced dose rate and improved temporal resolution while maintaining high spatial resolution. NFP is an excellent method for radiation sensitive samples. However, beam damage is a complex process with many experimental parameters to consider, such as beam size, photon energy and more. Björling et al. describe comprehensively the interplay between experimental parameters and free-radical formation in aqueous solutions^17^.

The spatial resolution of the scans was between 129 nm for the early scans, without significant beam damage, and 88 nm for the later scans, with significant beam damage, where we observe additionally to galvanic replacement a deposition of gold in the exposed areas of the sample. The increased size and thickness of the gold particles leads to an increased phase shift and thus an increased X-ray optical contrast, thereby improving the spatial resolution of the reconstructions of later scans. The resolution of all scans was close to or below the size of two pixels. Therefore, they can be described as pixel size limited when the resolution is considered in terms of the Nyquist-Shannon limit. In future experiments, the use of a detector with a smaller pixel size could improve the spatial resolution for similar temporal resolution and dose. Alternatively, an improved resolution could potentially be achieved by reducing the demagnified pixel size using a two-stage Fresnel propagator as described by Witte et al.^40^.

### In-situ imaging at highest spatial resolution

**Fig. 4:**
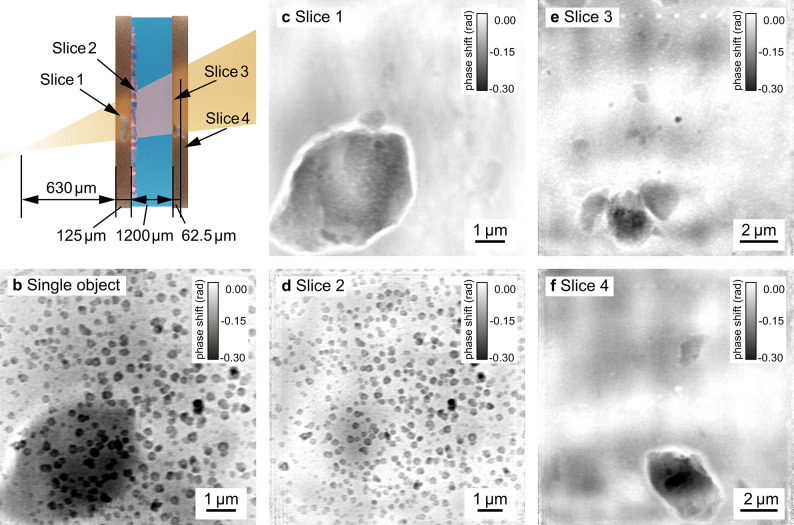
Imaging of nanoparticles growing in solution. (**a**) shows a schematic longitudinal cross-section of the chemical reactor with four distinct object planes (slices) in the direction of the X-ray beam. (**b**) A conventional single-slice reconstruction of the chemical reactor with nanoparticles. Applying the multi-slice approach allows to isolate four object planes corresponding to: (**c**) the upstream reactor’s window, (**d**) the inner side of the upstream window with nanoparticles, (**e**) the inner side of the downstream reactor’s window, and (**f**) the outer side of the downstream reactor’s window. (**c**), (**e**), and (**f**) show inclusions present in polyimide foils that otherwise obscure the view on the nanoparticles.

In a second in-situ series of the same reaction, our aim was to achieve imaging with an even higher spatial resolution. The reaction cell was moved to a position 630 µm downstream of the focal plane of the MLLs. By moving the sample closer to the focal plane, the magnification was increased by a factor of 4.75 to $M=5223$ and the pixel size in the first slice was reduced by the same factor to 14.4 nm. The reaction was running for 1.0 h when the scan shown in Fig. 4 was recorded. The distance between the upstream and downstream window was 1.2 mm, slightly larger than in the previous experiment. However, this is within the assembly tolerances of the reaction container.

Figure 4b shows a single-slice reconstruction, where the sample was treated as a single, optically thin object. A big inclusion in the polyimide foil is clearly visible in the background in the lower left corner. The polyimide foils often contain inclusions from additives used as slipping agents in the production process^41,42^ (see supplementary material Fig. S6), which compromise the reconstruction of the layer of interest containing the nanoparticles.

To optimize the resolution of the plane of interest and to remove any inclusions obscuring the view on the nanoparticles, we recovered four distinct slices (Fig. 4c–f) in a multi-slice reconstruction (see Table S2 in the supplementary material for specific reconstruction parameters). The two slices of the upstream window of the reaction cell (slice 1 and slice 2) are separated by 125 µm. The exit window is located 1200 µm downstream of the second slice and divided into two slices separated by 62.5 µm.

A distance of 630 µm between focal plane and sample may seem small to consider this measurement in the near-field imaging regime. The effective Fresnel number *F* for this scan is given by Eq. (4)^10^:

4 $$\begin{aligned} F=\frac{W^2}{\lambda z_\textrm{eff}}=126.5 \end{aligned}$$

with the extent of the illumination $W^2={2.2}\,\upmu \textrm{m}\times {2.5}\,\upmu \textrm{m}$, the wavelength $\lambda =$ 0.69 Å and the effective propagation distance $z_\textrm{eff}=$ 630 µm. Even though the effective Fresnel number for this measurement is considerably larger than unity, the scan can still be reconstructed with a far-field propagator between the last slice and the detector. However, the slice separation achieved with the near-field multi-slice reconstruction is superior to that of the far-field reconstruction (see supplementary material Fig. S4). The contribution from other slices remaining in the plane of interest is significantly smaller, showing that the Fresnel approximation models the measurement more accurately in this case. The plane with the nanoparticles (slice 2, Fig. 4d)﻿ shows only a weak shadow of the large inclusion from slice 1 compared to the single slice reconstruction. In order to correctly evaluate the reaction process, it is of great importance to obtain an undisturbed, quantitative image of the plane of interest, which we have achieved here in the multi-slice reconstruction. The large inclusions in slices 1, 3, and 4 are surrounded by a halo. This is a well-known artifact for strongly phase-shifting features in phase-contrast imaging^43–46^, resulting from the small modulation transfer of the low spatial frequencies.

The spatial resolution for slice 2, determined with Fourier ring correlation^47,48^, is 30 nm. This is close to the theoretical limit given by the focal spot size of the MLLs and below the resolution limit of two times the pixel size, given by the Nyquist criterion. The reconstruction can therefore be described as pixel size limited. The resolution for the other slices was 48 nm for the first slice, 84 nm for the third slice, and 130 nm for the fourth slice. The resolution of slice 1 is lower than slice 2 despite a smaller pixel size. This can be explained by a sparse sample plane for slice 1 with more than half of the scan points containing only little structural diversity and, hence, being more challenging to reconstruct. Details of the Fourier ring correlation analysis can be found in the supplementary material Fig. S3. For these spatial resolutions the slice thicknesses are all greater than the depth of field limit given by Eq. (2) (slice 1: 13 µm, slice 2: 8 µm, slice 3: 23 µm). Nevertheless, the separation of slice 3 and 4 does not appear to be perfect. The dark particle in the center of slice 3 appears as a white shadow in slice 4. The slice thickness seems to be close to the practical reconstruction limit. Hu et al. already describe that the actual thickness of the slices in X-ray NFP often has to be significantly larger than the theoretical limit given by Eq. (2)^24^. However, applying Eq. (1) with the constant $c=1$ seems to overestimate the limit for slice separation ($DOF=$ 102.6 µm).

We found that in the case of hard X-ray NFP, the experiment geometry must be well known at the time of measurement, to a much better degree than in the case of FFP experiments. The reconstruction is very sensitive to inaccuracies in defocus and interslice distance, as these have a strong impact on the effective Fresnel number.

In the scans following the one shown in Fig. 2, beam damage overshadows the reaction progress (see supplementary material Fig. S9). Again, in-situ imaging is a balancing act between spatial resolution and tolerable beam damage. Nevertheless, we have shown that NFP is competitive with FFP. Multi-slice NFP extends the capabilities of the method to the imaging of optically thick specimens, making it attractive for in-situ imaging.

## Conclusion

We demonstrated near-field multi-slice ptychography for in-situ imaging at high spatial resolution. This is the first X-ray NFP experiment with sub-50 nm spatial resolution.

In NFP, fewer scan points are needed for a given field of view compared to FFP, reducing the overhead time per scan to a minimum. Greater magnifications, which can be achieved by longer propagation distances, should enable larger illuminations without the loss of spatial resolution due to the large pixel size in the object plane in future experiments. Smaller detector pixels are another option to achieve smaller object pixel sizes. NFP with very large illuminations brings the method closer to full-field imaging and thus offers a significant reduction in measurement time. This makes the method attractive and suitable for studying systems with much better time resolution. With fully coherent flux at 4th generation synchrotron radiation sources, scan times of 1 min or less should be achievable for a field of view of comparable size to the results presented here.

MLLs have the highest *NA* for hard X rays from all state-of-the-art X-ray optics, providing exceptionally small focus sizes in this photon energy regime. The MLLs allow NFP with large illuminations relatively close to the focus, making them ideal for compact setups like the one used here. The high *NA* of MLLs also leads to a shallow depth of field. In our experiment, this allowed multi-slice reconstructions with slices separated by 62.5 µm, which is the smallest distance demonstrated so far in X-ray NFP. Further, the intrinsic structure of MLL-based illumination eliminated the need for a diffuser, even for sparse samples, such as nanoparticles.

For radiation-sensitive systems, in-situ near-field ptychography may be a better choice than near-focus scanning with far-field ptychography. The dose rate can be significantly reduced with NFP. However, radiation damage is a process that involves the interaction of many factors, and aqueous systems are particularly challenging in this regard. Diffusion rates and concentrations of free radicals as a function of the size of the irradiating beam would need to be systematically studied. The use of chemical inhibitors, such as radical scavengers, could be explored.

Regardless, NFP is a competitive method for in-situ imaging applications that could find widespread application with 4th generation light sources providing high temporal and spatial resolution.

## Methods

### Chemicals

Benzyl alcohol ($\ge 99.0 \%$), $\hbox {Cu(acac)}_2$ ($99.99\%$) and $\hbox {HAuCl}_4 \cdot \,\hbox {3H}_2$O ($\ge 99.9\%$ trace metal basis) were purchased from Sigma-Aldrich, and ethanol (absolute) for washing from VWR. All chemicals were used without further purification.

### Synthesis procedure

We adopted the synthetic procedure and the in-situ reactor reported elsewhere^39^. A 125 µm thick polyimide foil (Kapton HN, DuPont de Nemours Inc.) containing previously grown $\hbox {Cu}_2 \hbox {O}$ nanocubes was used in the entrance position of the in-situ reactor, while the exit window was empty. 1.4 mL Milli-Q ultrapure water with a resistivity of 18.2 M$\Omega$cm at 25 °C was filled into the reactor. At room temperature, a polytetrafluorethylene tube was inserted to dose 400 µL of a 20 mM aq. $\hbox {HAuCl}_4$ solution using a syringe pump (PHD Ultra, Harvard Apparatus, USA). The injection rate was set to 66 µL/h for sample 3 and 100 µL/h for sample 2. The galvanic replacement reaction is described by the following reaction equation^49^:

$$\begin{aligned} 1.5 \hbox {Cu}_{2}\hbox {O}_{\hbox {({s})}} + \hbox {AuCl}_{4\hbox {(aq)}}^{-} +3 \hbox {H}_{\hbox {(aq)}}^{+} \rightarrow \hbox {Au}_{\hbox {(s)}} + 3 \hbox {Cu}_{\hbox {(aq)}}^{2+} + 4 \hbox {Cl}_{\hbox {(aq)}}^{-} + 1.5 \hbox {H}_{2}\hbox {O} \end{aligned}$$

Au ions can be reduced in a competing reaction by solvated electrons and hydrogen radicals, products of the radiolysis of water by X-ray irradiation:

$$\begin{aligned} \begin{aligned}&\hbox {AuCl}_{4\hbox {(aq)}}^{-} + 3 \hbox {e}^{-}_\textrm{aq} \rightarrow \hbox {Au}^{0} + 4 \hbox {Cl}_{\hbox {(aq)}}^{-}\\&\hbox {AuCl}_{4\hbox {(aq)}}^{-} + 3 \hbox {H}{\cdot } \rightarrow \hbox {Au}^{0} + 3 \hbox {H}^{+} + 4 \hbox {Cl}_{\hbox {(aq)}}^{-} \end{aligned} \end{aligned}$$

This reaction can lead to additional deposition of Au on the two polyimide windows. Also other products of the hydrolysis, such as $\hbox{H}_2 \hbox{O}_2$, could act as reducing agents.

### Experimental setup

For the experiment we utilized the X-ray microscope PtyNAMi^50^ available at beamline P06 at PETRA III. The photon energy was set to 18.0 keV with a channel cut monochromator. To accommodate two multilayer Laue lenses, we used two hexapods which are components of the optics platform of the setup. The horizontally focusing lens was placed 160 µm downstream of the vertically focusing one. The lenses created a square aperture of 40 µm $\times$ 40 µm. Several millimeters downstream of the lenses we positioned a highly absorbing pinhole to block unwanted orders of diffraction. The resulting focal length of the MLLs at the selected photon energy equaled 13.5 mm. The numerical aperture (*NA*) of the MLLs was $1.48\cdot 10^{-3}$, which corresponds to a depth of focus of:

5 $$\begin{aligned} DOF_{MLL}=\frac{\lambda }{NA^{2}}=\frac{{68.88}\, \textrm{pm}}{(1.48\cdot 10^{-3})^{2}}={31.44}\, \upmu \textrm{m} \end{aligned}$$

The MLLs created an X-ray focal spot of 30 nm $\times$ 24 nm size (see supplementary material Fig. S9).

The 225 µm-thick polyimide foil was attached to a standard PtyNAMi sample holder. The foil sample and the reaction cell were positioned 0.63 mm to 3.00 mm downstream of the focus for NFP scans. The diffraction patterns were recorded by an in-vacuum single photon-counting Eiger X 4M detector at 3.29 m downstream of the sample.

### Scan parameters

The scans were recorded with a grid scan pattern. For each scan position, we introduced a random offset up to $30\%$ of the step size to avoid perfect-grid artifacts^51^.

**Sample 1** We placed the front surface of our sample (Slice 1) at a distance of 2.84 mm downstream of the X-ray focus. We scanned an area of 10 µm $\times$ 10 µm with a grid scan pattern of 31 $\times$ 31 scan points. The exposure time per scan point was 0.1 s. This geometry corresponds to an effective Fresnel number of $F=412$.

**Sample 2** The first slice was positioned at a distance of 3.00 mm downstream of the X-ray focus. We scanned an area of 10 µm $\times$ 10 µm with a grid scan pattern of 51 $\times$ 51 scan points. The exposure time per scan point was 0.2 s. The illuminating beam had a size of 8.7 µm $\times$ 9.3 µm and the effective Fresnel number for this scan was $F=392$.

**Sample 3** The first layer of the sample was at a distance of 0.63 mm from the X-ray focus. We scanned an area of 8 µm $\times$ 8 µm with a grid scan pattern of 51 $\times$ 51. The exposure time per scan point was 0.2 s. The Fresnel for this scan was $F=127$.

We list the detailed experimental and reconstruction parameters for each sample in the supplementary material Table S1 and Table S2.

### Lateral resolution determination

The lateral resolution of the scans was determined using Fourier ring correlation (FRC)^47,48^. For FRC, the reconstructions were carried out using one half of the scan points of the original data set. The scan positions were split in a way, that every second diffraction pattern was used, effectively mimicking a scanning step of twice the size in the vertical direction (fast scan axis). The half-bit criterion was used for the resolution determination since only half the diffraction patterns were used in each reconstruction. Details on the FRC for each scan are included in the supplementary material (Figs. S1, S2, and S3).

### Figures

All 3D renderings were created with *blender*^52^ and the figures were composed using *Adobe Illustrator*^53^ (version 28.1).

## Supplementary Information

Supplementary Information.

## Data Availability

The experimental datasets used for the results presented in this article are available at 10.5281/zenodo.15496659.

## References

[1] Pearce, A. K., Wilks, T. R., Arno, M. C. & O’Reilly, R. K. Synthesis and applications of anisotropic nanoparticles with precisely defined dimensions. *Nat. Rev. Chem.* **5**, 21–45. 10.1038/s41570-020-00232-7 (2020). 37118104 10.1038/s41570-020-00232-7

[2] Zhang, Z., Song, R., Cao, T. & Huang, W. Morphology-dependent structures and catalytic performances of Au nanostructures on O nanocrystals synthesized by galvanic replacement reaction. *J. Energy Chem.* **25**, 1086–1091. 10.1016/j.jechem.2016.09.012 (2016).

[3] Liao, H.-G., Cui, L., Whitelam, S. & Zheng, H. Real-time imaging of Fe nanorod growth in solution. *Science* **336**, 1011–1014. 10.1126/science.1219185 (2012). 22628649 10.1126/science.1219185

[4] Yoreo, J. J. D. In-situ liquid phase TEM observations of nucleation and growth processes. *Prog. Cryst. Growth Charact. Mater.* **62**, 69–88. 10.1016/j.pcrysgrow.2016.04.003 (2016).

[5] Wang, W., Yan, H., Anand, U. & Mirsaidov, U. Visualizing the conversion of metal-organic framework nanoparticles into hollow layered double hydroxide nanocages. *J. Am. Chem. Soc.* **143**, 1854–1862. 10.1021/jacs.0c10285 (2021). 33464886 10.1021/jacs.0c10285

[6] Ortiz Peña, N. et al. In situ liquid transmission electron microscopy reveals self-assembly-driven nucleation in radiolytic synthesis of iron oxide nanoparticles in organic media. *Nanoscale* **14**, 10950–10957. 10.1039/D2NR01511K (2022). 10.1039/d2nr01511k 35860928

[7] Sutter, E. A. & Sutter, P. W. In situ liquid cell electron microscopy of Ag-Au galvanic replacement reactions. *Nanoscale* **9**, 1271–1278. 10.1039/C6NR07293C (2017). 28054692 10.1039/c6nr07293c

[8] Chee, S. W., Tan, S. F., Baraissov, Z., Bosman, M. & Mirsaidov, U. Direct observation of the nanoscale Kirkendall effect during galvanic replacement reactions. *Nat. Commun.* **8**, 1224. 10.1038/s41467-017-01175-2 (2017). 29089478 10.1038/s41467-017-01175-2 PMC5663914

[9] Han, L. et al. Interrogation of 3d transition bimetallic nanocrystal nucleation and growth using in situ electron microscope and synchrotron X-ray techniques. *Nano Lett.* **24**, 7645–7653. 10.1021/acs.nanolett.4c01442 (2024). 38875704 10.1021/acs.nanolett.4c01442

[10] Stockmar, M. et al. Near-field ptychography: Phase retrieval for inline holography using a structured illumination. *Sci. Rep.* **3**, 1927. 10.1038/srep01927 (2013). 23722622 10.1038/srep01927 PMC3668322

[11] Shen, Q., Bazarov, I. & Thibault, P. Diffractive imaging of nonperiodic materials with future coherent X-ray sources. *J. Synchrotron Radiat.* **11**, 432–438. 10.1107/S0909049504016772 (2004). 15310961 10.1107/S0909049504016772

[12] Schropp, A. & Schroer, C. G. Dose requirements for resolving a given feature in an object by coherent x-ray diffraction imaging. *New J. Phys.* **12**, 035016. 10.1088/1367-2630/12/3/035016 (2010).

[13] Du, M., Gürsoy, D. & Jacobsen, C. Near, far, wherever you are: simulations on the dose efficiency of holographic and ptychographic coherent imaging. *J. Appl. Crystallogr.* **53**, 748–759. 10.1107/S1600576720005816 (2020). 32684890 10.1107/S1600576720005816 PMC7312132

[14] Xu, W., Ning, S. & Zhang, F. Numerical and experimental study of partial coherence for near-field and far-field ptychography. *Opt. Express* **29**, 40652–40667. 10.1364/OE.445978 (2021). 34809400 10.1364/OE.445978

[15] Clare, R. M., Stockmar, M., Dierolf, M., Zanette, I. & Pfeiffer, F. Characterization of near-field ptychography. *Opt. Express* **23**, 19728–19742. 10.1364/OE.23.019728 (2015). 26367630 10.1364/OE.23.019728

[16] Bras, W., Myles, D. A. A. & Felici, R. When X-rays alter the course of your experiments. *J. Phys. Condens. Matter* **33**, 423002. 10.1088/1361-648X/ac1767 (2021). 10.1088/1361-648X/ac1767 34298526

[17] Björling, A., Marçal, L. A. B., Arán-Ais, R. M. & Solla-Gullón, J. Chemical limits on X-ray nanobeam studies in water. *J. Phys. Chem. C* **127**, 13877–13885. 10.1021/acs.jpcc.3c02432 (2023).

[18] Shahmoradian, S. H. et al. Three-dimensional imaging of biological tissue by Cryo X-ray ptychography. *Sci. Rep.* **7**, 6291. 10.1038/s41598-017-05587-4 (2017). 28740127 10.1038/s41598-017-05587-4 PMC5524705

[19] Kadoma, Y. & Fujisawa, S. A comparative study of the radical-scavenging activity of the phenolcarboxylic acids caffeic acid, p-coumaric acid, chlorogenic acid and ferulic acid, with or without 2-mercaptoethanol, a thiol, using the induction period method. *Molecules* **13**, 2488–2499. 10.3390/molecules13102488 (2008). 18923340 10.3390/molecules13102488 PMC6244943

[20] Stachowski, T. R., Snell, M. E. & Snell, E. H. A SAXS-based approach to rationally evaluate radical scavengers—Toward eliminating radiation damage in solution and crystallographic studies. *J. Synchrotron Radiat.* **28**, 1309–1320. 10.1107/S1600577521004045 (2021). 34475280 10.1107/S1600577521004045 PMC8415334

[21] Howells, M. R. et al. An assessment of the resolution limitation due to radiation-damage in X-ray diffraction microscopy. *J. Electron Spectrosc. Relat. Phenom.* **170**, 4–12. 10.1016/j.elspec.2008.10.008 (2009). 10.1016/j.elspec.2008.10.008 PMC2867487 20463854

[22] Björling, A. et al. Ptychographic characterization of a coherent nanofocused X-ray beam. *Opt. Express* **28**, 5069. 10.1364/OE.386068 (2020). 32121735 10.1364/OE.386068

[23] Pan, A. & Yao, B. Three-dimensional space optimization for near-field ptychography. *Opt. Express* **27**, 5433–5446. 10.1364/OE.27.005433 (2019). 30876147 10.1364/OE.27.005433

[24] Hu, Z., Zhang, Y., Li, P., Batey, D. & Maiden, A. Near-field multi-slice ptychography: quantitative phase imaging of optically thick samples with visible light and X-rays. *Opt. Express* **31**, 15791–15809. 10.1364/OE.487002 (2023). 37157672 10.1364/OE.487002

[25] Hu, Z., Zhang, Y. & Maiden, A. Computational optical sectioning via near-field multi-slice ptychography. *Opt. Lett.* **49**, 4839–4842. 10.1364/OL.529190 (2024). 39207977 10.1364/OL.529190

[26] Öztürk, H. et al. Multi-slice ptychography with large numerical aperture multilayer Laue lenses. *Optica* **5**, 601–607. 10.1364/OPTICA.5.000601 (2018).

[27] Maiden, A. M., Humphry, M. J. & Rodenburg, J. M. Ptychographic transmission microscopy in three dimensions using a multi-slice approach. *J. Opt. Soc. Am. A* **29**, 1606–1614. 10.1364/JOSAA.29.001606 (2012). 10.1364/JOSAA.29.001606 23201876

[28] Stockmar, M. et al. X-ray nanotomography using near-field ptychography. *Opt. Express* **23**, 12720–12731. 10.1364/OE.23.012720 (2015). 26074526 10.1364/OE.23.012720

[29] Jacobsen, C. Relaxation of the Crowther criterion in multislice tomography. *Opt. Lett.* **43**, 4811–4814. 10.1364/OL.43.004811 (2018). 30272746 10.1364/OL.43.004811 PMC6410570

[30] Tsai, E. H. R., Usov, I., Diaz, A., Menzel, A. & Guizar-Sicairos, M. X-ray ptychography with extended depth of field. *Opt. Express* **24**, 29089–29108. 10.1364/OE.24.029089 (2016). 27958573 10.1364/OE.24.029089

[31] Cloetens, P., Barrett, R., Baruchel, J., Guigay, J.-P. & Schlenker, M. Phase objects in synchrotron radiation hard x-ray imaging. *J. Phys. D Appl. Phys.* **29**, 133–146. 10.1088/0022-3727/29/1/023 (1996).

[32] Maiden, A. M. & Rodenburg, J. M. An improved ptychographical phase retrieval algorithm for diffractive imaging. *Ultramicroscopy* **109**, 1256–1262. 10.1016/j.ultramic.2009.05.012 (2009). 19541420 10.1016/j.ultramic.2009.05.012

[33] Paganin, D. M. *Coherent X-Ray Optics* 1st edn. (Oxford University Press, 2006).

[34] Odstrčil, M., Lebugle, M., Guizar-Sicairos, M., David, C. & Holler, M. Towards optimized illumination for high-resolution ptychography. *Opt. Express* **27**, 14981. 10.1364/OE.27.014981 (2019). 31163938 10.1364/OE.27.014981

[35] Guizar-Sicairos, M. et al. Role of the illumination spatial-frequency spectrum for ptychography. *Phys. Rev. B* **86**, 100103. 10.1103/PhysRevB.86.100103 (2012).

[36] van der Walt, S. et al. scikit-image: image processing in Python. *PeerJ* **2**, e453. 10.7717/peerj.453 (2014). 25024921 10.7717/peerj.453 PMC4081273

[37] Kahnt, M. et al. Multi-slice ptychography enables high-resolution measurements in extended chemical reactors. *Sci. Rep.* **11**, 1500. 10.1038/s41598-020-80926-6 (2021). 33452343 10.1038/s41598-020-80926-6 PMC7810740

[38] Pogany, A., Gao, D. & Wilkins, S. W. Contrast and resolution in imaging with a microfocus x-ray source. *Rev. Sci. Instrum.* **68**, 2774–2782. 10.1063/1.1148194 (1997).

[39] Grote, L. et al. Multimodal imaging of cubic Cu2O@Au nanocage formation via galvanic replacement using X-ray ptychography and nano diffraction. *Sci. Rep.* **13**, 318. 10.1038/s41598-022-26877-6 (2023). 36609430 10.1038/s41598-022-26877-6 PMC9823101

[40] Witte, S., Pelekanidis, A. & Eikema, K. Propagators with user-defined object-plane pixel size for ptychography. *Optica Open*10.1364/opticaopen.28163246.v1 (2025).

[41] Arnquist, I. J., Beck, C., di Vacri, M. L., Harouaka, K. & Saldanha, R. Ultra-low radioactivity Kapton and copper-Kapton laminates. *Nucl. Instrum. Methods Phys. Res. Sect. A* **959**, 163573. 10.1016/j.nima.2020.163573 (2020).

[42] Fang, Y. et al. A bio-enabled maximally mild layer-by-layer Kapton surface modification approach for the fabrication of all-inkjet-printed flexible electronic devices. *Sci. Rep.* **6**, 39909. 10.1038/srep39909 (2016). 28008987 10.1038/srep39909 PMC5180237

[43] Zernike, F. How I discovered phase contrast. *Science* **121**, 345–349. 10.1126/SCIENCE.121.3141.345 (1955). 13237991 10.1126/science.121.3141.345

[44] Chang, H. et al. Advanced denoising for X-ray ptychography. *Opt. Express* **27**, 10395–10418. 10.1364/OE.27.010395 (2019). 31052900 10.1364/OE.27.010395

[45] Ozsoy-Keskinbora, C., Boothroyd, C. B., Dunin-Borkowski, R. E., van Aken, P. A. & Koch, C. T. Hybridization approach to in-line and off-axis (electron) holography for superior resolution and phase sensitivity. *Sci. Rep.* **4**, 7020. 10.1038/srep07020 (2014). 25387480 10.1038/srep07020 PMC4228327

[46] Kandel, M. E., Fanous, M., Best-Popescu, C. & Popescu, G. Real-time halo correction in phase contrast imaging. *Biomed. Opt. Express* **9**, 623. 10.1364/BOE.9.000623 (2018). 29552399 10.1364/BOE.9.000623 PMC5854064

[47] Banterle, N., Bui, K. H., Lemke, E. A. & Beck, M. Fourier ring correlation as a resolution criterion for super-resolution microscopy. *J. Struct. Biol.* **183**, 363–367. 10.1016/j.jsb.2013.05.004 (2013). 23684965 10.1016/j.jsb.2013.05.004

[48] van Heel, M. & Schatz, M. Fourier shell correlation threshold criteria. *J. Struct. Biol.* **151**, 250–262. 10.1016/j.jsb.2005.05.009 (2005). 16125414 10.1016/j.jsb.2005.05.009

[49] Lowe, J. M. & Coridan, R. H. Mechanistic control of a galvanic replacement reaction on cuprous oxide. *Nanoscale Adv.* **1**, 1343–1350. 10.1039/C8NA00396C (2019).

[50] Schropp, A. et al. PtyNAMi: ptychographic nano-analytical microscope. *J. Appl. Crystallogr.* **53**, 957–971. 10.1107/S1600576720008420 (2020). 32788903 10.1107/S1600576720008420 PMC7401781

[51] Odstrčil, M., Holler, M. & Guizar-Sicairos, M. Arbitrary-path fly-scan ptychography. *Opt. Express* **26**, 12585. 10.1364/oe.26.012585 (2018). 29801297 10.1364/OE.26.012585

[52] B. O. Community. Blender—A 3D Modeling and Rendering Package (2024).

[53] Adobe Inc. Adobe Illustrator (2024).

