# Supplementary material for: X-ray near-field multi-slice ptychography for in-situ imaging
Source: Sci Rep. 2025 Sep 1;15:32150. doi: 10.1038/s41598-025-15610-8 (PMC12402461; doi:10.1038/s41598-025-15610-8)
Supplement: Supplementary file 1 — Supplementary Information. [file 41598_2025_15610_MOESM1_ESM.pdf]

# Supplementary material - X-ray near-field multi-slice ptychography with multilayer Laue lenses for in-situ imaging

**Sina Röper[1,2,+], Karolina Stachnik[1,+,*], Jonas Voss[1], Sarah-Alexandra Hussak[1,2], Mattias Åstrand[3], Lukas Grote[1], Felix Wittwer[4,5], Martin Seyrich[2], Sven Niese[6], Peter Gawlitza[7], Ulrich Vogt[3], Christian G. Schroer[1,2,8], Dorota Koziej[1], and Andreas Schropp[2,*]**

[1]Center for Hybrid Nanostructures, Institute for Nanostructure and Solid-State Physics, University of Hamburg, Luruper Chaussee 149, 22761, Hamburg, Germany
[2]Center for X-ray and Nano Science CXNS, Deutsches Elektronen-Synchrotron DESY, Notkestraße 85, 22607, Hamburg, Germany
[3]KTH Royal Institute of Technology, Department of Applied Physics, Bio-Opto-Nano Physics, Albanova University Center, 106 91 Stockholm, Sweden
[4]NERSC, Lawrence Berkeley National Laboratory, Berkeley, CA 94720, USA
[5]Current address: Deutsches Elektronen-Synchrotron DESY, Hamburg, Germany
[6]AXO DRESDEN GmbH, Gasanstaltstraße 8b, 01237 Dresden, Germany
[7]Fraunhofer Institute for Material and Beam Technology (IWS), Winterbergstrasse 28, 01277 Dresden, Germany
[8]Helmholtz Imaging, Deutsches Elektronen-Synchrotron DESY, Notkestraße 85, 22607, Hamburg, Germany
*Email: karolina.stachnik@xray-lens.de; andreas.schropp@xray-lens.de
+these authors contributed equally to this work

## Supplementary Method 1: Near field multi-slice ptychography

### Implementation of the Fresnel scaling theorem

For near-field multi-slice ptychography Fresnel scaling has to be implemented in the 3PIE[1] reconstruction algorithm. The cone beam is converted to a parallel beam geometry by scaling of the distances and pixel sizes. The following adaptations were done:

The user defines the number of slices $N$ and the real distances from one slice to the next $z_{n\to(n+1)}$. The algorithm begins by calculating the magnification from the slice closest to focus, $n = 0$, to the detector (index $d$):

$$M_{0\to d} = 1 + \frac{z_{0\to d}}{z_{\text{focus}\to 0}} \tag{1}$$

This magnification is used to calculate the object pixel size $\Delta x_0$ of the slice $n = 0$, as given by:

$$\Delta x_0 = \frac{\Delta x_d}{M_{0\to d}}, \tag{2}$$

where $\Delta x_d$ is the pixel size of the detector. For the propagation between slices, the inter-slice magnification between slices $n$ and $n+1$ is calculated for all slices:

$$M_{n\to(n+1)} = 1 + \frac{z_{n\to(n+1)}}{z_{\text{focus}\to n}} \tag{3}$$

From this, the effective propagation distance $z_{\text{eff}}$ between the two slices follows:

$$z_{\text{eff},n\to(n+1)} = \frac{z_{n\to(n+1)}}{M_{n\to(n+1)}} \tag{4}$$

The object pixel size of a subsequent slice $n+1$, $\Delta x_{n+1}$, is calculated by scaling the pixel size of the current slice $n$ with the inter-slice magnification.

$$\Delta x_{n+1} = \Delta x_n \cdot M_{n\to(n+1)} \tag{5}$$

where the pixel size of the first slice $\Delta x_0$ is used as the starting point. The algorithm works with discretized probe positions, calculated from the real-space positions and the pixel size. With the object pixel size changing from one slice to the next, the

pixel-valued positions need to be calculated for each slice separately, even though the real-space positions are not changed. Effectively, there are two changes to 3PIE algorithm for the application in the near-field ptychography: firstly, the near-field propagator is not only used in between slices but also for the propagation from the final slice to the detector and back. Secondly, Fresnel scaling theorem is applied to all object pixel sizes, propagation distances and discretized probe positions.

## Supplementary Method 2: Experiment and reconstruction

### Experiment and reconstruction parameters

Table S1 summarizes the experimental parameters for the scans of each figure 2, 3, and 4. Table S2 lists the settings for each reconstruction.

| Parameter | Fig. 2 | Fig. 3 | Fig. 4 |
|---|---|---|---|
| Photon energy [keV] | 18.0 | 18.0 | 18.0 |
| X-ray beam prefocusing [on / off] | on | off | on |
| Focus-to-sample distance [mm] | 2.84 | 3.0 | 0.63 |
| Grid-scan step size [nm] | 323 | 196 | 157 |
| Additive random jitter interval [%] | $\pm 30$ | $\pm 30$ | $\pm 30$ |
| Exposure time [s] | 0.1 | 0.2 | 0.2 |
| Sample-to-detector distance [m] | 3.29 | 3.29 | 3.29 |
| Detector pixel size [$\mu$m] | 75 | 75 | 75 |

**Table S1. Experiment parameters.** Parameters used for the ptychograms shown in Fig. 2, 3, and 4.

| Parameter | Fig. 2c | Fig. 2e-f | Fig. 3c-h | Fig. 4b | Fig. 4c-f |
|---|---|---|---|---|---|
| Reconstruction mode | single-slice | multi-slice | multi-slice | single-slice | multi-slice |
| Number of slices | 1 | 2 | 2 | 1 | 4 |
| Focus - slice 1 distance [mm] | 2.84 | 2.84 | 3.0 | 0.63 | 0.63 |
| Slice 1 pixel size [nm] | 64.7 | 64.7 | 68.3 | 14.4 | 14.4 |
| Slice 1 refinement delay [iterations] | – | 100 | 50 | – | 15 |
| Slice 1 - Slice 2 distance [mm] | – | 0.225 | 1.0 | – | 0.125 |
| Slice 2 pixel size [nm] | – | 69.8 | 91.1 | – | 17.2 |
| Slice 2 refinement delay [iterations] | – | 0 | 0 | – | 30 |
| Slice 2 - Slice 3 distance [mm] | – | – | – | – | 1.2 |
| Slice 3 pixel size [nm] | – | – | – | – | 44.6 |
| Slice 3 refinement delay [iterations] | – | – | – | – | 5 |
| Slice 3 - Slice 4 distance [mm] | – | – | – | – | 0.0625 |
| Slice 4 pixel size [nm] | – | – | – | – | 46.0 |
| Slice 4 refinement delay [iterations] | – | – | – | – | 0 |
| Cropping area on detector [pixels] | $512 \times 512$ | $512 \times 512$ | $512 \times 512$ | $512 \times 512$ | $512 \times 512$ |
| Reconstruction engine | ePIE[2] | 3PIE[1] | 3PIE[1] | ePIE[2] | 3PIE[1] |
| Number of iterations | 1000 | 1000 | 5000 | 1000 | 1000 |
| Position refinement | on[3] | on[3] | off | on[3] | on[3] |

**Table S2. Near-field ptychographic reconstruction parameters.** Parameters for the single-slice and multi-slice reconstructions of Fig. 2, 3, and 4.

### *Fourier ring correlation of complementary ptychographic sub-datasets*

For each multi-slice reconstruction the resolution was determined with Fourier ring correlation[4,5]. For each scan the resolution of the layer(s) of interest was close to or below two pixels. The spatial resolution in this experiment can, therefore, be called pixel size limited. The evaluation for the reconstructions shown in Fig. 2 are shown in Fig S1, for Fig. 3 in Fig. S2, and for Fig. 4 in Fig. S3. All plots were created with *matplotlib*[6] (version 3.9.2[7]).

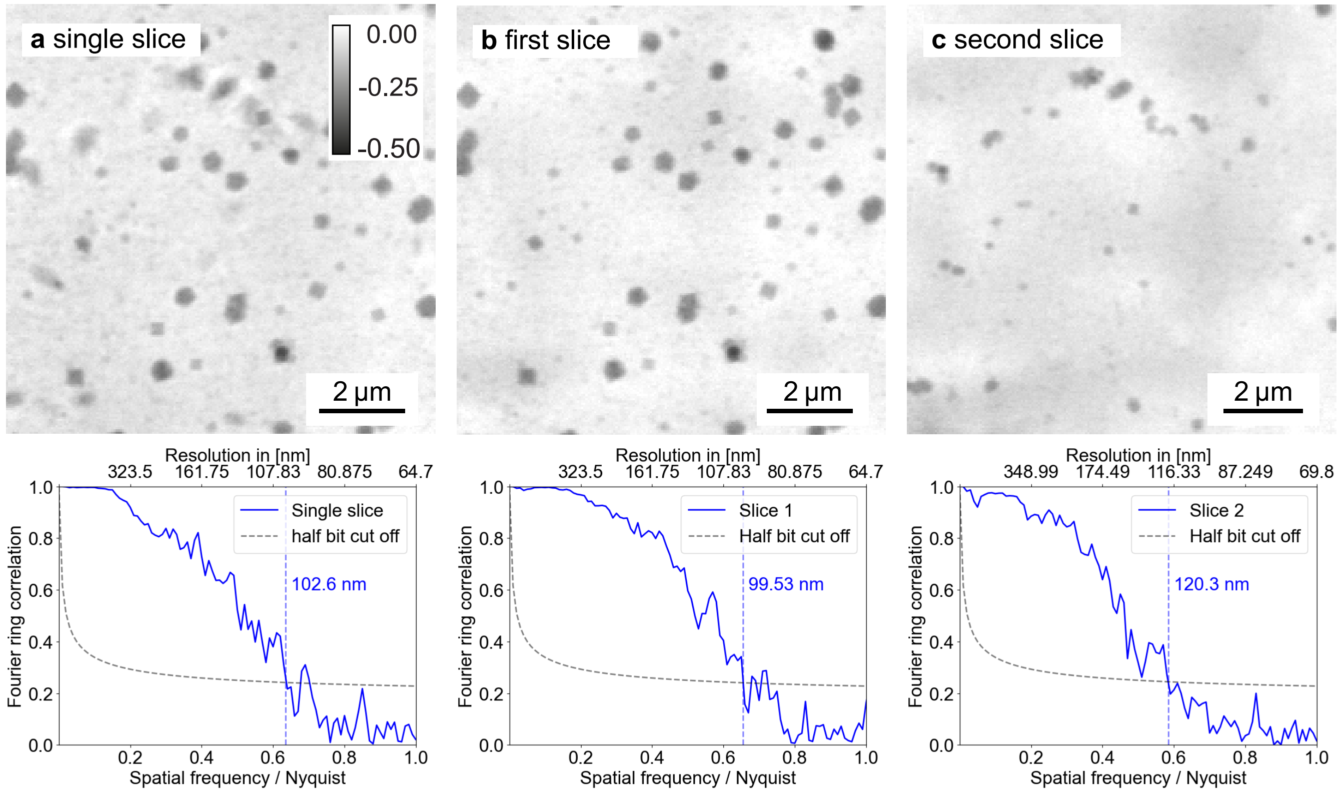


**Figure S1. Spatial resolution determination for $Cu_2O$ cubes on both sides of a 225 $\mu$m thick foil. a** Ptychographic reconstructions of the object as a single slice. The gray scale indicates the phase shift in radians. For the Fourier ring correlation the reconstructions were carried out using one half of the scan points of the original data set. The dotted line in the bottom plot shows the half-bit cut off. The resolution is determined to be 103 nm for the single slice. The resolution for the first slice was calculated to be 100 nm **b** and 120 nm for the second slice **c**. This corresponds to 1.4 px and 1.7 px, respectively. Considering, that the spatial resolution, taking into account the Nyquist criterion, can not be better than two pixels, we conclude that the achieved resolution is for both slices pixel size limited.

## Comparison near-field and far-field multislice reconstruction

The scan shown in Fig. 4 can be reconstructed as a near-field or a far-field ptychogram. The measurement was carried out at the edge between near- and far-field regime. The results of both reconstructions is shown in Fig. S4. The reconstructions were run with the same distances and number of iterations. Only few reconstruction parameters were adapted, all other parameters were the same as used for the near-field reconstruction. The far-field reconstruction was carried out with a three times virtually enlarged illumination[8], which was needed for sufficient sampling of the spatial frequencies. The propagation between the last slice and the detector was modeled with a fast Fourier transform, and the slices were initialized in a different order (starting iteration for slice 1: 20, slice 2: 0, slice3: 50, slice 4: 80). The illumination was initialized as a gaussian beam with a curved wavefront, whereas it was flat for the near-field reconstruction.

The near-field multi-slice reconstruction does separate the features of each slice well, whereas in the far-field multi-slice contributions from slice 1 remain in slice 2 and the same for contributions from slice 4 in slice 3. To get a reliable image of the plane of interest (slice 2), an unobscurred reconstruction result is crucial, which in this case can be better achieved with the near-field multi-slice reconstruction.

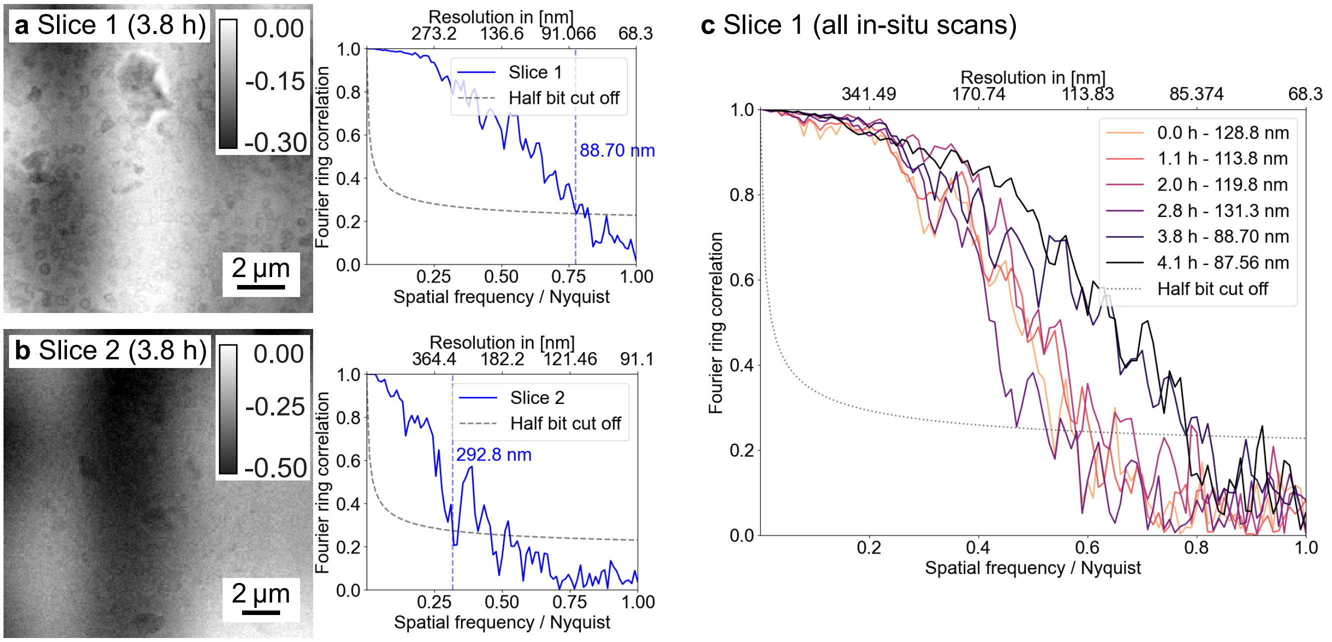


**Figure S2.** **Spatial resolution determination for the in-situ series a** Exemplary ptychographic reconstruction of the first slice of the scan taken after 3.8 h of the reaction start. Each image was reconstructed using one half of the scan points of the original data set. The gray scale indicates the phase shift in radians. The resolution is determined to be 88.7 nm, which corresponds to 1.3 px. The resolution can, therefore, be seen as pixel size limited. **b** The resolution of the second slice of the same scan was determined to be 293 nm. **c** Overview of the spatial resolution of the first slice for each scan of the in-situ series. The resolution improved over the course of the reaction starting with 129 nm (1.9 px) to 88 nm (1.3 px) at the end of the reaction.

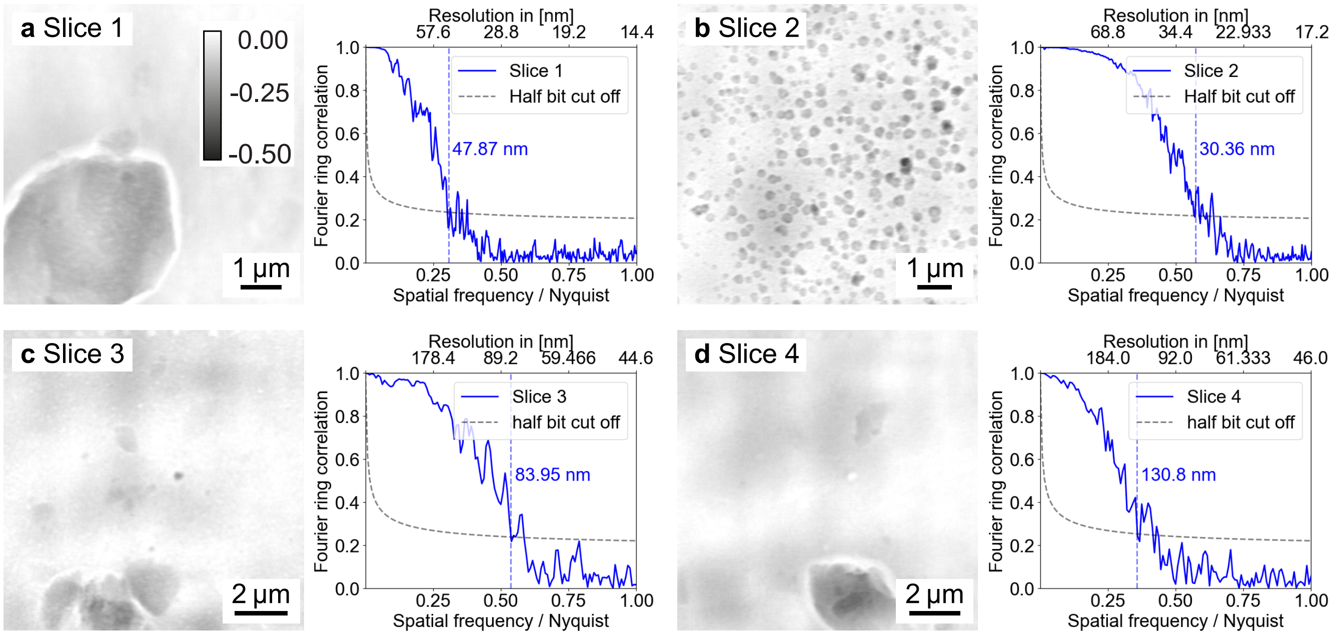


**Figure S3.** **Spatial resolution determination for the reconstructions of the in-situ cell. a** Multi-slice reconstruction of the object. The gray scale indicates the phase shift in radians. For the Fourier ring correlation the reconstructions were carried out using one half of the scan points of the original data set. The dotted line in the bottom plot shows the half-bit cut off. The resolution is determined to be 48 nm for the first slice. The spatial resolution for the second slice **b** was calculated to be 30 nm, for the third slice **c** 84 nm, and for the fourth slice **d** 131 nm.

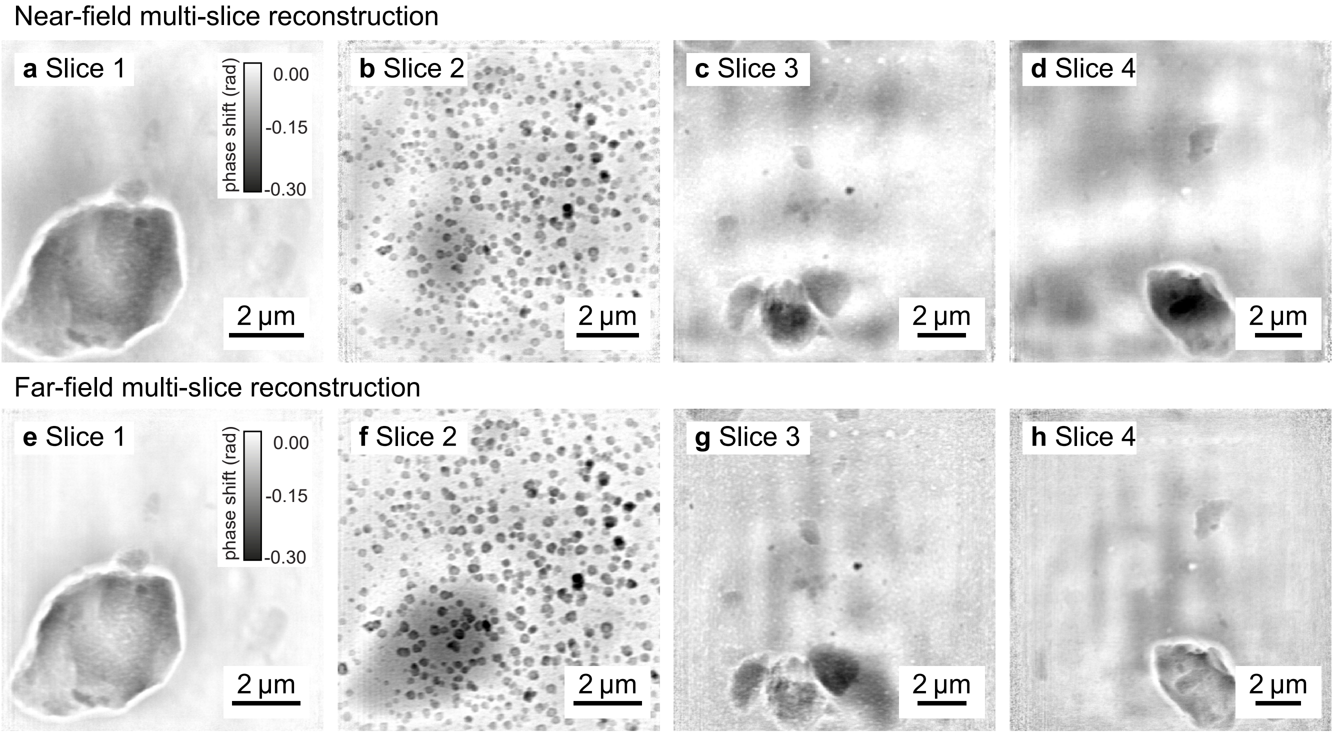


**Figure S4. Comparison of a near-field and far-field multi-slice reconstruction. a - d** Near-field reconstructions of the four slices. **e - h** Far-field reconstructions of the same scan. The big inclusion of slice 1 casts a big shadow in the second slice in the far-field reconstruction (**f**), while there is only a very faint shadow in the near-field reconstruction (**b**). The same effect can be seen in slice 3, where a shadow from the particle of slice 4 remains in the far-field reconstruction, but is considerably weaker in the near-field reconstruction.

## Supplementary Method 3: Synthesis

### In-situ reaction cell

The reaction cell shown in Fig. S5 was used for both in-situ measurements. Details on the cell can be found in Grote et al.[9]

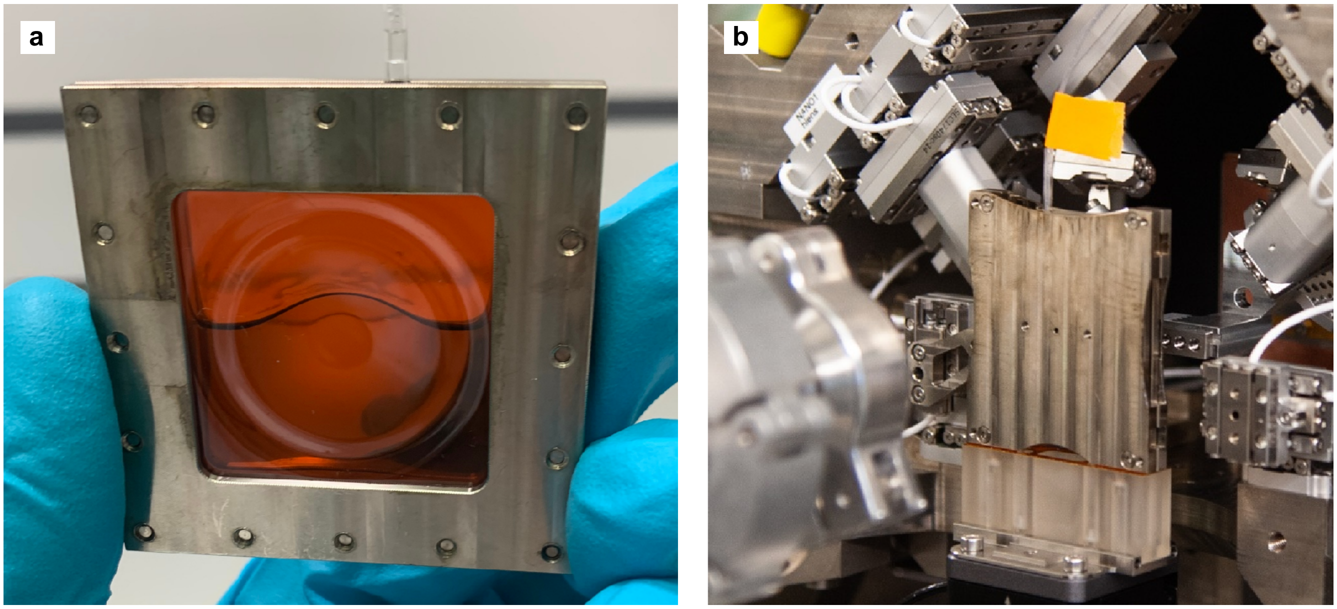


**Figure S5. In-situ reaction cell. a** Inner part of the reaction cell containing the reaction solution. Two polyimide foils and a PTFE frame (hidden behind the metal part), that separates the foils, are enclosed in a metal frame. The reaction solution can be added through a glass capillary at the top. **b** The complete reaction cell at the beamline mounted on the scanning stage.

### Inclusions in the polyimide foils

The polyimide foils, used as the substrate for the synthesis, often contain inclusions. These inclusions are most likely a slip additive introduced during the production process of the foils. The inclusions are reported to be dicalcium phosphate[10] or calcium carbonate[11]. Fig. S6 shows light microscope images of a polyimide foil of the same type that was used for the synthesis.

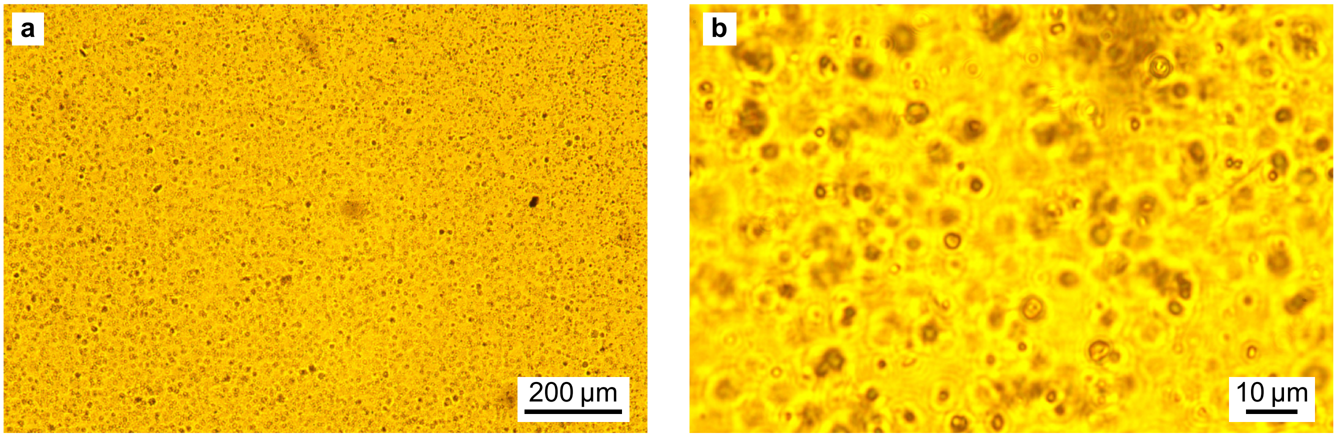


**Figure S6. Inclusions in the polyimide foil. a** A light microscope image shows that dicalcium phosphate inclusions are wide spread over the entire polyimide foil. **b** Inclusions are present in different depths of the foil with varying size between 1 μm and 8 μm. Particles embedded deeper in the foil than the depth resolution appear blurred.

## Galvanic replacement reaction

Fig. S7 shows the sample used for the in-situ reaction shown in Fig. 3 before and after the reaction. $Cu_2O$ cubes with an edge length of 150 nm to 250 nm were synthesized on a polyimide foil. The $Cu_2O$ cubes underwent a galvanic replacement reaction with Au leading to the formation of hollow Au nanocages. In the area that was exposed to the X-ray beam a layer of Au formed. This growth of Au beyond the formation of the Au shells can be attributed to beam damage.

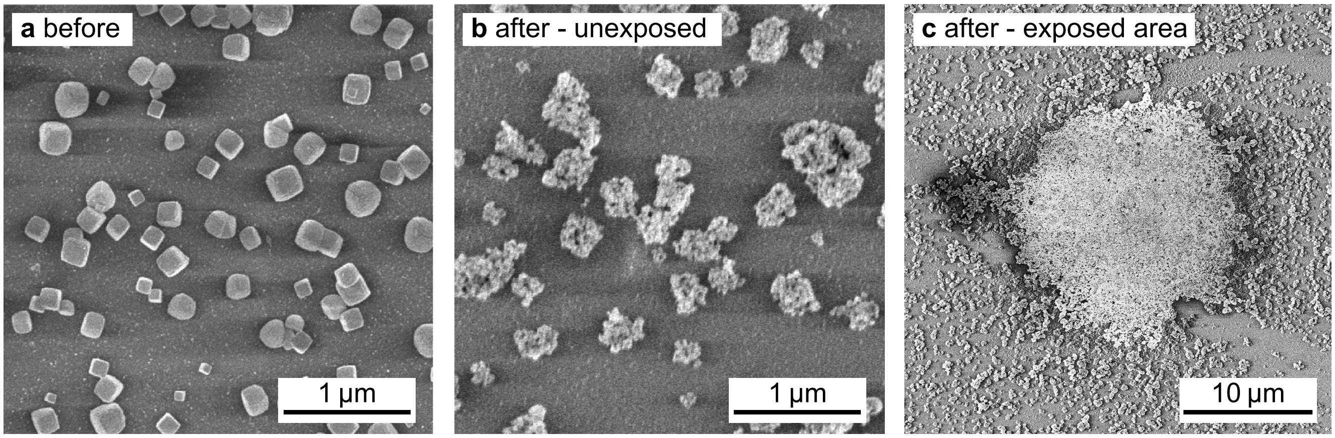


**Figure S7. Scanning electron microscopy images of the sample before and after the galvanic replacement reaction. a** Polyimide foil with attached $Cu_2O$ cubes, that was used in the in-situ series shown in Fig. 3. **b** An area of the same sample after the galvanic replacement reaction with Au that was not exposed to the X-ray beam. **c** In the area that was continuously scanned a layer of Au formed. Images were acquired with a Regulus8220 SEM (Hitachi, Japan) at an acceleration voltage of 0.3 kV.

## Beam damage induced Au growth on the back foil

Fig. S8 shows the beam induced deposition of Au on the back side of the reaction container in the in-situ series shown in Fig. 3. The formation of Au on the empty foil is not observed in ex-situ laboratory experiments as it is not related to the galvanic replacement reaction, but is instead caused by beam damage.

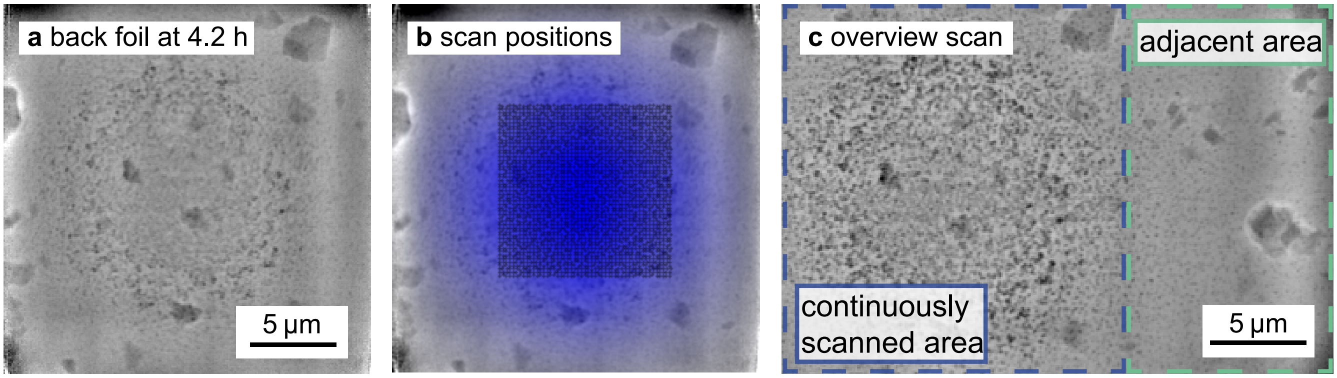


**Figure S8. Beam damage in the in-situ series shown in Fig. 3. a** shows the initially empty back foil of the reaction container at 4.2 h. Normally, one would expect the foil to remain empty apart from the inclusions in the polyimide foil. **b** indicates the scan positions with the black dots overlayed on the scan shown in **a**. The blue color indicates the intensity of the beam over the field of view. The beam intensity is calculated by summing up the amplitude of the illumination over all scan positions. **c** is an overview scan at the end of the reaction. The left side of the scan was continuously scanned for the duration of the reaction. The right side was scanned only once for the overview scan. It is apparent that in the continuously scanned area of the sample a significant amount of gold was deposited. The right side remained mostly empty as it would be expected in experiments without X-ray beam exposure. The overlay in **b** was created with *matplotlib*[6] (version 3.9.2[7]).

### Progression of beam damage induced Au growth

The high resolution in-situ series shown in Fig. 4 suffered from heavy beam damage. The normal course of the reaction was overshadowed by beam induced growth of elemental Au on the foils (Fig. S9). The Au growth is driven by the reduction of $Au^{3+}_{(aq)}$ by solvated electrons or H-radicals, which are created through radiolysis in the X-ray beam.

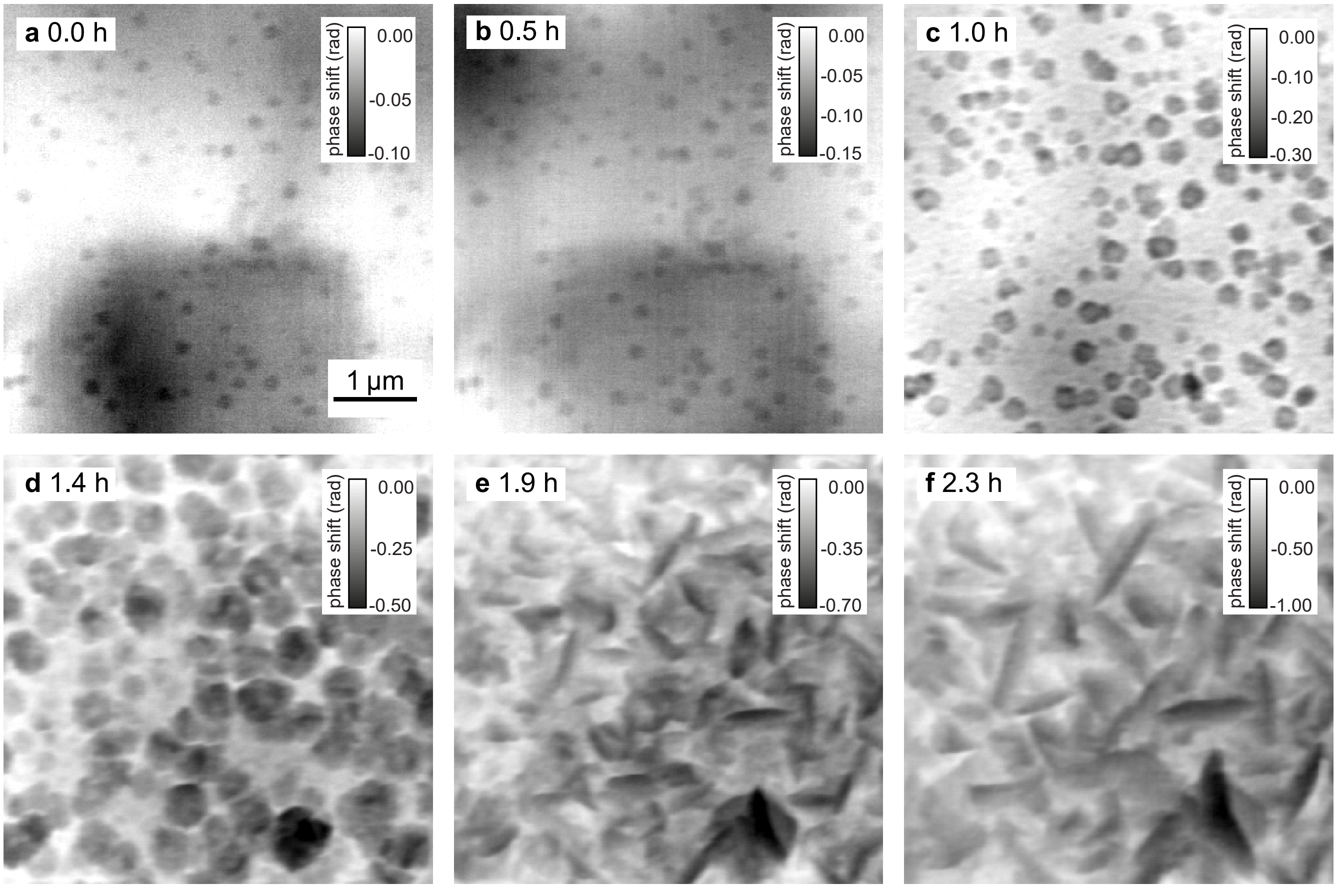


**Figure S9. In situ series corresponding to Fig. 4 a-f** Show reconstructions of the inner side of the upstream window (slice 2) on which the $Cu_2O$ nanocubes underwent a galvanic replacement reaction with Au. From **d** onwards the reaction does not resemble the result of laboratory experiments anymore. Micrometer long Au needles grow on the polyimide foils.

## Supplementary Method 4: Optics

### Multilayer Laue lenses - X-ray beam caustic

The multilayer Laue lenses created a focus of 30 nm × 24 nm size (S10c). The beam caustic is shown in S10a and b. A far-field ptychographic scan of a Siemens star test pattern was used for the X-ray beam characterization.

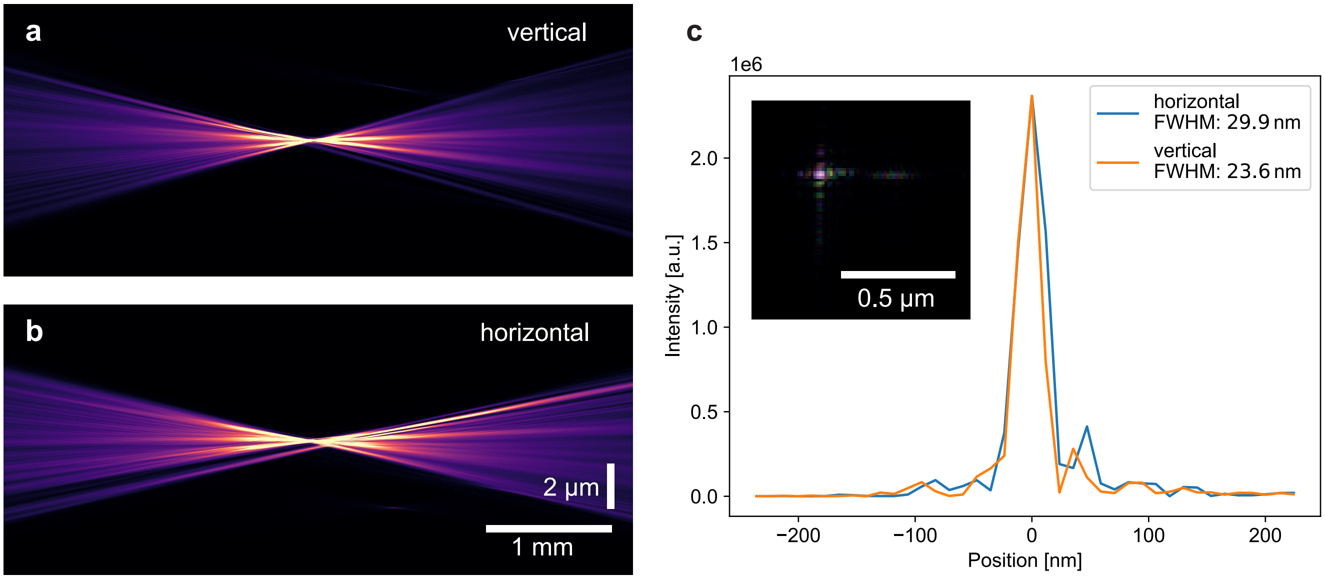


**Figure S10. Characterizaiton of the X-ray beam focused by multilayer Laue lenses.** Beam caustic in vertical (**a**) and horizontal (**b**) direction. **c** Inlay shows the beam in the focal plane. The full width half maximum (FWHM) was determined to be 29.9 nm × 23.6 nm. The plot was created with *matplotlib*[6] (version 3.9.2[7]).

## References


1. Maiden, A. M., Humphry, M. J. & Rodenburg, J. M. Ptychographic transmission microscopy in three dimensions using a multi-slice approach. *J. Opt. Soc. Am. A* **29**, 1606–1614, DOI: 10.1364/JOSAA.29.001606 (2012).
2. Maiden, A. M. & Rodenburg, J. M. An improved ptychographical phase retrieval algorithm for diffractive imaging. *Ultramicroscopy* **109**, 1256–1262, DOI: 10.1016/j.ultramic.2009.05.012 (2009).
3. Schropp, A. *et al.* Full spatial characterization of a nanofocused x-ray free-electron laser beam by ptychographic imaging. *Sci. Reports* **3**, 1633, DOI: 10.1038/srep01633 (2013).
4. Banterle, N., Bui, K. H., Lemke, E. A. & Beck, M. Fourier ring correlation as a resolution criterion for super-resolution microscopy. *J. Struct. Biol.* **183**, 363–367, DOI: 10.1016/j.jsb.2013.05.004 (2013).
5. van Heel, M. & Schatz, M. Fourier shell correlation threshold criteria. *J. Struct. Biol.* **151**, 250–262, DOI: 10.1016/j.jsb.2005.05.009 (2005).
6. Hunter, J. D. Matplotlib: A 2D Graphics Environment. *Comput. Sci. & Eng.* **9**, 90–95, DOI: 10.1109/MCSE.2007.55 (2007).
7. Matplotlib development team. Matplotlib: Visualization with Python v3.9.2 (2024).
8. Wittwer, F. *et al.* Ptychography with a Virtually Enlarged Illumination. *Microsc. Microanal.* **24**, 48–49, DOI: 10.1017/S1431927618012667 (2018).
9. Grote, L. *et al.* Multimodal imaging of cubic $Cu_2O$@Au nanocage formation via galvanic replacement using X-ray ptychography and nano diffraction. *Sci. Reports* **13**, 318, DOI: 10.1038/s41598-022-26877-6 (2023).
10. Arnquist, I. J., Beck, C., di Vacri, M. L., Harouaka, K. & Saldanha, R. Ultra-low radioactivity Kapton and copper-Kapton laminates. *Nucl. Instruments Methods Phys. Res. Sect. A: Accel. Spectrometers, Detect. Assoc. Equip.* **959**, 163573, DOI: 10.1016/j.nima.2020.163573 (2020).
11. Fang, Y. *et al.* A bio-enabled maximally mild layer-by-layer Kapton surface modification approach for the fabrication of all-inkjet-printed flexible electronic devices. *Sci. Reports* **6**, 39909, DOI: 10.1038/srep39909 (2016).
